# Supplementary material for: Building a synthesis of economic costs of biological invasions in New Zealand
Source: PeerJ. 2022 Aug 15;10:e13580. doi: 10.7717/peerj.13580 (PMC9387519; doi:10.7717/peerj.13580)
Supplement: Supplemental Information 1 [file peerj-10-13580-s001.pdf]

Te hanga kōtuitanga o ngā utu ōhanga o ngā urutomonga koiora i Aotearoa

He wae matua ngā urutomonga koiora ki te huringa o te taiao i ngā paranuku ā-tāngata, e he nui hoki te utu ā-ōhanga ki ngā rāngai, wāhanga katoa o te hapori whānui me ōna pūnaha rauropi. Ina noa nei kua kōtuituia ngā utu o ētahi o ngā whenua e whakamahi ana i te pātengi raraunga InvaCost, he kohikohinga hou o nā tata nei, heoi anō, a Aotearoa — tētahi whenua rongonui mō te āhua o tana whakahaere i ngā kararehe urutomo - tae noa ki tēnei wā kāore anō kia tirohia. I konei ka tātaria ngā utu o te tūkinotanga ā-ōhanga, me ngā utu whakahaere i utua mō ngā urutomonga ā-koiora i Aotearoa mai i te tau 1968 ki te tau 2020. Hui katoa, ko te nui o te utu i pūrongotia mō tēnei wāhanga 50 tau, ko te US$69 piriona (NZ$97 piriona), me te āhua US$9 piriona o tēnei e whakaarohia ana, he tino tika, ka mutu i kitea whānuitia ēnei utu (ā-matapae). E whai pānga ana te nuinga (82%) o ēnei utu ā-ōhanga i kitea ki te tūkinotanga, kāore i tino nui te pūtea i whakapaua ki te mahi whakahaere (18%). E piki haere ana ngā utu i pūrongotia i roto i te wā, kei te āhua US$120 miriona te utu toharite o te tūkinotanga i ia tau, ka mutu, e hipa noa ana i te whakapaunga mahi whakahaere i ngā ngahuru tau katoa. Ina tauwhāitihia, ko te nuinga o ngā utu i pūrongotia kua ahu mai i ngā tipu ahupapa me ngā kararehe, ka mutu, e kawea ana te nuinga o ngā tūkinotanga e ngā ahumahi tuatahi pērā ki te mahi ahuwhenua me te mahi ngahere. E whai pānga kē ana te nuinga o ngā utu whakahaere ki ngā wawaotanga e ngā mana me te hunga whai pānga. Mō te wāhi ki ētahi atu whenua i roto i te pātengi raraunga InvaCost, i kītea he nui noa ake te whakapaunga a Aotearoa i tērā i whakaarohia mai i tana Hua ā-Motu Katoa ki ngā utu whakahaere i mua, i muri hoki i te urutomonga. Heoi anō, kei te ngaro i te whakapae o ngā utu tūkino i matapaetia ētahi kararehe urutomo he kaha te tūkino e mōhiotia ana ā-kaiao (ā-ōhanga), ka mutu, kāore e pūrongotia ana ngā utu whakahaere mō ētahi o ngā kararehe puihi, me ngā tukumate ahuwhenua rānei. I runga i te mōhio ki ēnei ngaronga mō ngā kaiurutomo ka kaha tūkino pea, e whakatenatena ana mātou i te whakapikinga o te pūrongotanga utu ki te taumata ā-motu, tae atu ki te whakapaitanga ake o te whai wāhitanga a te iwi whānui mai i te whakapikinga o te whai wāhitanga mai me te whakamatihikotanga o ngā pūkete, ina koa i ngā rāngai oha-pori, momo taiao hoki kāore i tino arohia atu. He mea whakapuaki anō hoki tēnei i te hiranga o te whakapau pūtea ki ngā mahi whakahaere hei haukoti i ngā mahi tūkino puta noa i ngā rāngai katoa hei ngā rā e tū mai nei.
