# Supplementary material for: Building a synthesis of economic costs of biological invasions in New Zealand
Source: PeerJ. 2022 Aug 15;10:e13580. doi: 10.7717/peerj.13580 (PMC9387519; doi:10.7717/peerj.13580)

**Supplementary Material 1: tables and figures**

**Table S1:** Summary of all unique species entries (either single or multi-species or genus level) for New Zealand recorded in the InvaCost 4.0 database (accessed December 2020).

| Species | Common Name | Costing Type | Spatial Scale(s) | Raw cost (USD m, 2017) | DB appearances | Citation(s) |
| --- | --- | --- | --- | --- | --- | --- |
| Terrestrial Environments | |  |  |  |  |  |
| *Canis lupus/Felis catus/Mustela erminea/Mustela furo* | Feral dog/Feral cat/stoat/ferret | Management | Unit (ha) | 1.17 | 1 | Busch and Cullen 2008 |
| *Capra hircus* | Feral goat | Management | Country, site, unit (park) | 24.10 | 4 | DOC 2002, Martins et al. 2006, Forsyth et al. 2014 |
| *Capra hircus/Cervus elaphus/*  *Rupicapra rupicapra* | Feral goat/Red deer/Alpine chamois | Management | Site | 0.07 | 2 | Forsyth et al. 2014 |
| *Capra hircus/Felis catus/*  *Oryctolagus cuniculus/*  *Sus scrofa/Vulpes vulpes* | Feral goat/Feral cat/European rabbit/Feral pig/Red fox | Management | Unit (km) | 0.05 | 1 | Long and Robley 2004 |
| *Capra hircus/Sus scrofa* | Feral pig/Feral goat | Management | Unit (km) | <0.01 | 1 | Long and Robley 2004 |
| *Carduus nutan* | Thistle | Management, Damage | Unit (ha) | <0.01 | 2 | Moore et al. 1989 |
| *Ceratitis capitata* | Mediterranean fruit fly | Management | Country | 3.78 | 1 | Allwood et al. 2002 |
| *Ceratitis spp.* | Fruit fly | Management | Country | 1.97 | 1 | MPI 2013, Stephenson et al. 2003 |
| *Cervus sp.* | Deer | Management | Site, unit (km), unit (hour) | 0.10 | 3 | Fraser et al. 2003, Nugent and Choquenot 2004 |
| *Cirsium arvense* | Californian thistle | Management, damage, mixed | Country, unit (ha) | 510.23 | 4 | Chalak et al. 2008, Chalak et al. 2011, Bourdot et al. 2016 |
| *Clematis vitalba* | Old man's beard | Management | Country, site | 2.56 | 4 | Greer et al. 1990 |
| *Cytisus scoparius* | Scotch broom | Management | Country | 2.58 | 1 | Jarvis et al. 2006 |
| N/A | diverse terrestrial species | Management, damage | Country, site, unit (ha) | 60774.73 | 45 | Monsanto 1984, Bourdot et al. 2007, Towns et al. 1993, Martins et al. 2006, McFadden and Greene 1994, Goldson and Suckling (Eds.) 2003, Towns et al. 1993, Turner et al. 2004, Parkes and Murphy 2003, Krause et al. 2001, DOC 2002, Williams and Jones 2002, Clout 2002, Barlow and Goldson 2002, Environmental Risk Management Authority 2007, Vessey 2010, Griffith 2011, Russell et al. 2015, Verlarde et al. 2015, Parkes et al. 2017, Senator and Rozenberg 2016, Ferguson et al. 2019, Hawkins et al.2011, Holmes et al. 2015 |
| *Erinaceus europaeus/Felis catus/Mus musculus/Mustela erminea/Oryctolagus cuniculus/Rattus norvegicus/Rattus exulans/Rattus rattus* | hedgehog/cat/house mouse/stoat/rabbit/Brown rat/Polynesian rat/black rat | management | site | 3.24 | 1 | Griffiths et al. 2015 |
| *Felis catus/Rodent sp.* | Cats/Rodents | Management | Site | 0.95 | 1 | Campbell et al. 2011 |
| *Felis catus* | Feral Cat | Management,Management | Unit (2 Landholders),Site | 65.41 | 2 | Glen et al. 2016, Parkes et al. 2017 |
| *Felis catus/Rattus rattus/Trichosurus vulpecula* | Cat/Black rat/Common brushtail possum | Management | Site | 39.89 | 1 | Beaven 2008 |
| *Felix catus/Rattus sp.* | Cat/Rats | Management | Site | 0.09 | 1 | Martins et al. 2006 |
| *Gallirallus australis/Rattus sp.* | Weka/Rats | Management | Site | 0.06 | 1 | Martins et al. 2006 |
| *Hemitragus jemlahicus* | Himalayan tahr | Management | Country | 22.82 | 2 | Flueck 2010 |
| *Hyphantria cunea* | Fall Webworm | Management, damage | Country | 44.52 | 2 | Gordh and McKirdy 2014 |
| *Linepithema humile* | Argentine ant | Management | Site | 0.30 | 1 | Green 2020 |
| *Listronotus bonariensis* | Argentine stem weevil | Damage | Country | 284.48 | 3 | Prestidge et al. 1991, Tomasetto et al. 2017 |
| *Lymantria dispar* | Gypsy moth | Management, damage | Country | 531.68 | 5 | Turner et al. 2004, Salamolard et al. 2008, Gordh and McKirdy 2014 |
| *Metopolophium dirhodum* | Rose-grass aphid | Mixed | Country | 12.18 | 1 | Grundy 1989 |
| *Mus musculus/Mustela erminea/Trichosurus vulpecula/Rattus rattus* | House mouse/Black rat/Common brushtail possum/Stoat | Management | Country | 22736.83 | 1 | Parkes et al. 2017 |
| *Mus musculus/Rattus rattus* | House mouse/Black rat | Management | Site | 0.07 | 1 | Broome 2009 |
| *Mus musculus/Trichosurus vulpecula* | House mouse/Common brushtail possum | Management | Unit (km) | 0.17 | 1 | Karori Reservoir Wildlife Sanctuary Steering Committee 1994 |
| *Mustela erminea* | Stoat | Management | Country, Unit (ha) | 1.59 | 3 | Parkes and Murphy 2003, Warburton and Gormley 2015, Norton and Warburton 2015 |
| *Mustela erminea/Rattus rattus/Trichosurus vulpecula* | stoat/black rat/possum | Management | unit (ha) | < 0.01 | 1 | Norton and Warburton 2015 |
| *Mustela erminea/Trichosurus vulpecula* | stoat/common brushtail possum | Management | country | 4.99 | 1 | Cabinet 2016 |
| *Mustela furo* | ferret | Management | site | 0.03 | 1 | Anderson et al. 2013 |
| *Mustela furo/Trichosurus vulpecula* | ferret/Common brushtail possum | Management | Country, site | 34.55 | 2 | Anderson et al. 2013, Animal Health Board 2002 |
| *Nasonovia ribisnigri* | Lettuce Aphid | Damage | Country | 0.13 | 1 | Stufkens et al. 2002 |
| *Nassella neesiana* | Chilean needle grass | Management | Site | 0.19 | 1 | Harris 2010 |
| *Nassella trichotoma* | Nassella tussock | Management | Country, site | 315.36 | 3 | Lamoureaux et al. 2015, Denne 1998 |
| *Orgyia thyellina* | White-spotted tussock moth | Management, damage | Country, site | 218.94 | 7 | Gordh and McKirdy 2014, Turner et al. 2004, Wittenberg and Cock 2001 |
| *Oryctolagus cuniculus* | European rabbit | Management, damage | Site, unit (ha) | 208.31 | 6 | King 2017, Wodzicki 1950, Hunt and Rasheed 1991, Martin 1994, Parkes and Murphy 2003, Latham et al. 2016 |
| *Oryctolagus cuniculus/Rattus sp.* | European rabbit/Rats | Management | Site | 0.02 | 1 | Martins et al. 2006 |
| *Passer domesticus* | House sparrow | Damage | Country | 0.20 | 1 | Dawson 1970 |
| *Pieris brassicae* | Large white butterfly | Management | Site | 3.14 | 1 | Brown, Phillips, Broome, Green, Toft, Walker 2019 |
| *Pilosella officinarum* | Mouse-ear hawk weed and king devil hawk weed | Management, damage | Country | 6.72 | 2 | Grundy 1989 |
| *Ranunculus acris* | Giant buttercup | Management, Damage | Country, site | 1089.40 | 9 | Saunders et al. 2017, Bourdôt et al. 2003 |
| *Pilosella sp.* | hawkweeds | damage | country | 5.975 | 1 | Scott 1984 |
| *Rattus exulans* | Polynesian rat | Management | Site, unit (ha) | 0.60 | 5 | McFadden and Towns 1991, McFadden 1992, Broome 2008 |
| *Rattus exulans/Rattus norvegicus* | Polynesian rat/Brown rat | Management | Site, unit (ha) | 2.59 | 6 | Broome 2009, Towns and Broome 2003 |
| *Rattus norvegicus* | Brown rat | Management | Site, unit (ha) | 3.09 | 6 | Taylor and Thomas 1989, Towns and Broome 2003, Broome 2009 |
| *Rattus rattus* | Black rat | management | Unit (ha) | < 0.01 | 3 | Norton and Warburton 2015;  Warburton and Gormley 2015 |
| *Rattus sp.* | Rats | management | site | 1.911 | 13 | Martins et al. 2006 |
| *Rattus sp./Trichosurus vulpecula* | Rats/Common brushtail possum | management | unit (ha) | < 0.01 | 3 | Clapperton and Day 2001 |
| *Rattus spp./Trichosurus vulpecula* | Rats/Common brushtail possum | management | Site, unit (forest) | 0.25 | 2 | Morgan et al. 2015 |
| *Rhopalosiphum padi* | Bird cherry-oat aphid | Damage | Site | 2.88 | 1 | Bicknell and Greer 1999 |
| *Rosa rubiginosa* | sweet brier | Management, mixed | country | 5.77 | 2 | Grundy 1990 |
| *Setaria helvola* | yellow bristle grass | damage | unit (island) | 221.60 | 1 | Tozer et al. 2014 |
| *Solenopsis invicta* | Red Imported Fire Ant | Management, damage, mixed | Country, site, unit (ha) | 589.60 | 15 | Gutrich et al. 2007, Olsen et al. 2005, Wang et al. 2020, Gruber et al. 2021, Anonymous 2001 |
| *Sus scrofa* | Feral pig | Management | Site | 58.97 | 1 | Parkes et al. 2017 |
| *Teia anartoides* | Painted apple moth | Management, damage | Country, site | 716.95 | 8 | Gordh and McKirdy 2014, New Zealand Herald 2001, Goldson et al. 2005, Controller and Auditor-General of New Zealand 2002, Turner et al. 2004 |
| *Thaumetopoea pityocampa* | Pine processionary moth | Management, damage | Country | 1257.90 | 2 | Kriticos et al. 2013 |
| *Trichosurus vulpecula* | Common brushtail possum | Management, damage, mixed | Country, site, unit (ha) | 393.55 | 20 | Livingstone et al 2015, Cowan 1992, Martins et al 2006, Anonymous 1986, Cowan 1991, Hutchings et al. 2013, Bertram 1999, Choquenot and Parkes 2000, Hutchings et al. 2013, DOC 2002, Livingstone et al. 2015, Anderson et al. 2013, Warburton et al. 2012, Tait et al. 2017, Parkes et al. 2017, Gormley et al. 2015 |
| *Ulex europaeus* | gorse | management | country | 34.54 | 2 | Sandrey 1985 |
| *Uraba lugens* | Gum leaf skeletoniser | Management, damage | Country | 216.22 | 3 | Gordh and McKirdy 2014 |
| *Varroa destructor* | Varroa mite | Management, damage | Country, site | 658.12 | 4 | Wittenberg and Cock 2001, Anonymous 2003, Simpson 2003 |
| *Varroa jacobsoni* | Varroa mite | damage | country | < 0.01 | 1 | Matthews 2005 |
| *Vespula spp* | Wasps | Damage | Site | 95.92 | 1 | MacIntyre, Hellstrom 2005 |
| Diverse/Unspecified Environments (includes aquatic/terrestrial and semi-aquatic environment types) | |  |  |  |  |  |
| N/A | N/A | Management, damage | Country | 1639.991 | 2 | Mumford 2002, Russell et al. 2015 |
| N/A | Animals | Management | Unit (species) | 0.04 | 1 | Jenkins 2013 |
| N/A | Animals/plants | Damage | Country | 1242.56 | 1 | Nimmo-Bell 2009 |
| *Trichosurus vulpecula*/unspecified | Common brushtail possum, mustelids, rodents | Management | Country | 20.43 | 1 | Kopf et al. 2017 |
| N/A | Microorganisms and invertebrates | Management | Country | 5.30 | 1 | Goldson and Suckling (Eds.) 2003 |
| N/A | Diverse macroinvertebrates | Management | Country | 2.33 | 1 | Goldson and Suckling (Eds.) 2003 |
| N/A | Diverse/unspecified semi-aquatic | Management | Site | 46.66 | 1 | Egeter et al. 2019 |
| N/A | Diverse weeds and pests | Management | Country | 638.34 | 2 | Goldson and Suckling (Eds.) 2003 |
| N/A | Unspecified | Damage | Country | 2565.85 | 2 | Paini et al. 2016 |
| N/A | Predators | Management | Unit (ha, km) | 13.16 | 4 | Clapperton and Day 2001 |
| *Aedes camptorhynchus* | Southern saltmarsh mosquito | Management | Country | 47.28 | 1 | Champion 2018 |
| N/A | Stoats and other pests | Management | Site, unit (ha) | 0.23 | 4 | Clapperton and Day 2001 |
| Aquatic Environments | |  |  |  |  |  |
| *Styela clava* | Asian tunicate | Damage, | Site,Site | 24.23 | 2 | Soliman and Inglis 2017 |
| *Didymosphenia geminata* | Didymo | Damage | Country | 8.04 | 1 | Beville et al. 2012 |
| N/A | Diverse aquatic weeds | Management | Country | 0.74 | 1 | Goldson and Suckling (Eds.) 2003 |
| N/A | Diverse/unspecified | Management | Country | 0.95 | 1 | Schaffelke and Hewitt 2007 |
| *Sabella spallanzanii* | Feather duster worm | Damage | Site | 15.63 | 2 | Soliman and Inglis 2017 |
| N/A | Unspecified | Management | Country | 9.29 | 2 | Hewitt and Bauckham 2004, Schaffelke and Hewitt 2007 |
| *Undaria pinnatfida* | Wakame | Management, | Unit (sunken trawler) | 14.63 | 3 | Wotton et al. 2004 |

**Table S2:** Costs (billions of 2017 US$ and NZ$) of biological invasions in New Zealand across environment types from the InvaCost v4.0 database using only robust costs (i.e. highly reliable and observed).

| **Environment** | **Cost (US$ b)** | **Cost (NZ$ b)** | ***n*** |
| --- | --- | --- | --- |
| Terrestrial | 3.22 | 4.51 | 229 |
| Aquatic | 0.10 | 0.14 | 105 |
| Diverse/Unspecified | 5.46 | 7.64 | 21 |
| Semi-aquatic | 0.05 | 0.07 | 13 |

**Table S3:** Costs (2017 USD and NZD) of biological invasions in New Zealand across socioeconomic sectors from the InvaCost v4.0 database using only robust costs (i.e. highly reliable and observed).

| **Sector** | **Cost (US$ b)** | **Cost (NZ$ b)** | ***n*** |
| --- | --- | --- | --- |
| Authorities and Stakeholders | 1.39 | 1.95 | 184 |
| Authorities and Stakeholders/Health | <0.01 | <0.01 | 12 |
| Agriculture | 2.31 | 3.23 | 93 |
| Unspecified | 3.19 | 4.47 | 3 |
| Agriculture/Forestry | 1.58 | 2.21 | 1 |
| Forestry | 0.26 | 0.36 | 5 |
| Health | 0.06 | 0.09 | 14 |
| Fishery/Public and social welfare | <0.01 | <0.01 | 1 |
| Agriculture/Authorities-stakeholders | <0.01 | <0.01 | 1 |
| Public and social welfare | 0.01 | 0.02 | 48 |
| Environment | <0.01 | <0.01 | 6 |

**Table S4:** Total expenditure over 1968 - 2020 across management cost types from the InvaCost v4.0 database using only robust costs  (i.e. highly reliable and observed).

| **Management Type** | **Cost (US$ b)** | **Cost (NZ$ b)** | ***n*** |
| --- | --- | --- | --- |
| Pre-invasion management | 0.07 | 0.10 | 23 |
| Post-invasion management | 1.41 | 1.97 | 196 |
| Knowledge/funding | 0.12 | 0.17 | 19 |
| Mixed | <0.01 | <0.01 | 2 |

Figure S1: Scatterplots of GDP versus a) pre-invasion management costs, b) post-invasion management costs and c) damage costs for all countries represented within the InvaCost database for which robust cost estimates exist. New Zealand is highlighted as the red dot in each case, showing higher management investment than predicted from its GDP. The dashed lines represent the linear model, with both variables on a natural-log scale.


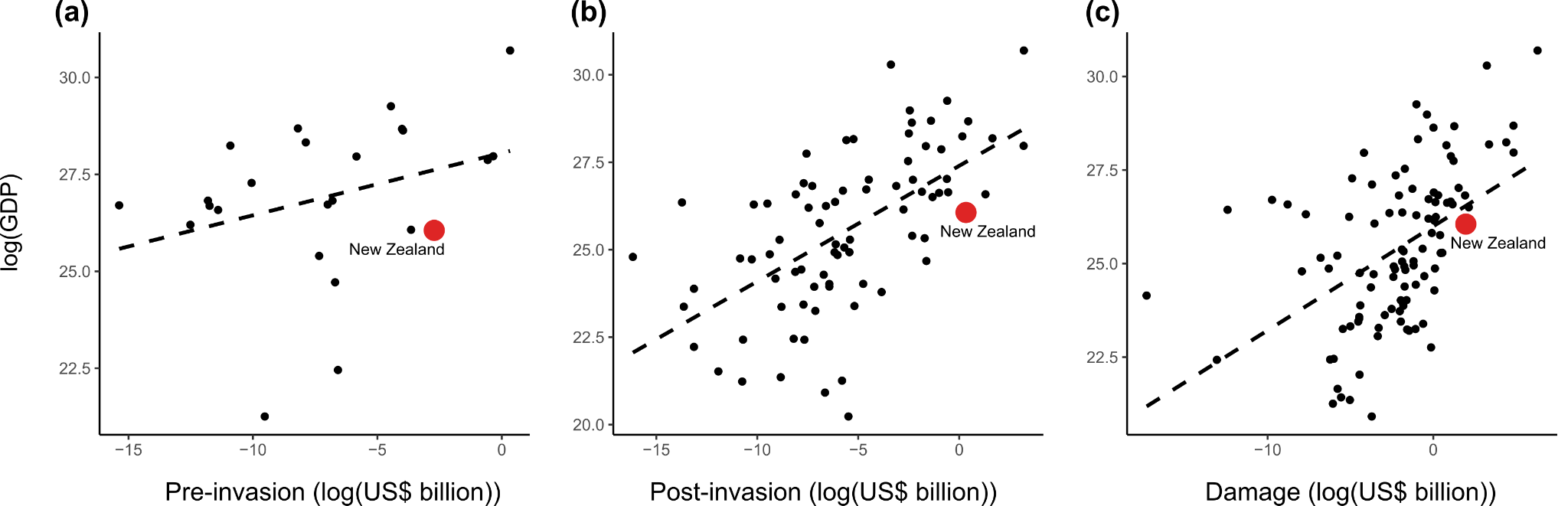

Supplement: Supplemental Information 2 [file peerj-10-13580-s002.docx]
